# The Role of Urinary Biomarkers as Diagnostic and Prognostic Predictors of Acute Kidney Injury Associated With Vancomycin

**DOI:** 10.3389/fphar.2021.705636

**Published:** 2021-09-23

**Authors:** Durval Sampaio de Souza Garms, Karina Zanchetta Cardoso Eid, Emmanuel A. Burdmann, Lia Junqueira Marçal, Leila Antonângelo, Adriano dos Santos, Daniela Ponce

**Affiliations:** ^1^ Department of Internal Medicine, University of São Paulo State, São Paulo, Brazil; ^2^ LIM 12, Nephrology Discipline of University of São Paulo, São Paulo, Brazil; ^3^ Clinics Hospital Pharmacy, Botucatu School of Medicine, São Paulo, Brazil

**Keywords:** acute kidney injury, nephrotoxicity, vancomycin, biomarkers, IL-18, NGAL, IGFBP7, TIMPS-2

## Abstract

**Introduction:** The incidence of acute kidney injury (AKI) related to vancomycin is variable, and several risk factors related to the treatment and patients may explain the nephrotoxicity. The role of urinary biomarkers in AKI related to vancomycin is unknown.

**Objective:** The aim of this study was to evaluate the role of urinary IL-18, KIM-1, NGAL, TIMP-2, and IGFBP7 as diagnostic and prognostic predictors of AKI related to vancomycin.

**Methods:** A prospective cohort study of patients receiving vancomycin and admitted to wards of a public university hospital from July 2019 to May 2020 was performed. We excluded patients that had AKI before starting vancomycin, hemodynamic instability, inability to collect urine, and chronic kidney disease stage 5.

**Results:** Ninety-four patients were included, and the prevalence of AKI was 24.5%, while the general mortality was 8.7%. AKI occurred 11 ± 2 days after the first vancomycin dose. The most frequent KDIGO stage was 1 (61%). There was no difference between patients who developed and did not develop AKI due to gender, length of hospital stay, dose, and time of vancomycin use. Logistic regression identified age (OR 6.6, CI 1.16–38.22, *p* = 0.03), plasmatic vancomycin concentrations between 96 and 144 h (OR 1.18, CI 1.04-1.40, *p* = 0.04), and urinary NGAL levels between 96 and 144 h (OR 1.123, CI 1.096–1.290, *p* = 0.03) as predictors of AKI. The time of vancomycin use (OR 4.61, CI 1.11–22.02, *p* = 0.03), higher plasmatic vancomycin concentrations between 192 and 240 h (OR 1.02, CI 0.98–1.06, *p* = 0.26), and higher cell cycle arrest urinary biomarkers TIMP-2 multiplied by IGFBP-7 between 144 and 192 h (OR 1.33, CI 1.10–1.62, *p* = 0.02; OR 1.19, CI 1.09–1.39, *p* = 0.04, respectively) were identified as prognostic factors for non-recovery of kidney function at discharge.

**Conclusion:** AKI related to vancomycin was frequent in patients hospitalized in wards. Age, plasmatic vancomycin concentrations, and NGAL between 96 and 144 h were identified as predictors of AKI related to vancomycin use. Plasmatic vancomycin concentrations and urinary NGAL were predictors of AKI diagnosis within the next 5 days. The urinary biomarkers of cell cycle arrest TIMP-2 and IGFBP-7 and the duration of vancomycin use were associated with non-recovery of kidney function at hospital discharge moment.

## Introduction

Vancomycin is a widely used drug in intensive care units (ICUs), as oxacillin-resistant gram-positive cocci are the main infectious etiological agents. The main polemic points on vancomycin relate to its efficacy and safety. Regarding efficacy, the concern is the alteration in pharmacokinetics, usually related to the distribution, metabolism, and elimination of the drug, which can lead to subtherapeutic concentrations, leading to the emergence of bacterial resistance due to the inefficacy of the treatment and consequently leading to higher mortality. Regarding safety, emphasis has been given to the main side effect of the medication—nephrotoxicity—which can aggravate the clinical picture and contribute to unfavorable outcomes and occurs 4–8 days after starting to use. The pathophysiological mechanisms are not yet fully understood, but undoubtedly it is ongoing with a consequent worsening of the prognosis of these patients (Zamoner et al., 2020).

The incidence of vancomycin nephrotoxicity varies widely between different studies, reaching up to 40% and there are different factors related to the patient and/or the drug that can accelerate the nephrotoxicity. Among the factors associated with the patient, we highlight, mainly, advanced age, sepsis, dehydration, and reduced kidney function, while among the risk factors related to the drug are the concomitant administration with other drugs such as loop diuretics, amphotericin B, aminoglycosides, vasopressors, and intravenous contrast media; the longer duration of treatment and the high plasmatic vancomycin concentration (Elyasi et al., 2012; Zamoner et al., 2020).

It is recommended that the monitoring of the plasmatic concentration of vancomycin be performed in patients at high risk for renal dysfunction, critically ill, in those who receive high vancomycin doses and/or in treatment for more than 5 days.

Some authors, however, still question whether the high plasmatic vancomycin concentrations are the cause or the consequence of AKI (Elyasi et al., 2012; Hanrahan et al., 2014).

Changes in plasmatic creatinine and urine output are still the standards for recognizing AKI. However, it is known that creatinine and urine output are influenced by factors such as hypercatabolism, drugs, hydration status, muscle mass, gender, and age, which can delay the diagnosis of AKI. In addition, AKI may be present even in the absence of an increase in plasma creatinine due to renal functional reserve or tubular creatinine secretion. These limitations for the use of serum creatinine spurred research to discover new, more sensitive, specific, and early biomarkers in the diagnosis and prognosis of AKI, including the tubular lesion markers kidney injury molecule-1 (KIM-1), interleukin 18 (IL-18), neutrophil gelatinase-associated lipocalin (NGAL), tissue inhibitor of metalloproteinases 2 (TIMP-2), and insulin-like growth factor binding protein 7 (IGFBP-7) (Andreucci et al., 2017; Schrezenmeier et al., 2017).

Today there is no perfect biomarker of AKI. Each biomarker is not completely specific for AKI. This is reflected by the imperfect test characteristics of each biomarker with the best AUCROCs ranging between 0.75 and 0.85. The pathophysiologic basis for this lack of sensitivity and specificity is only partially understood. For instance, NGAL and IL-18 are known to be produced in immune cells and display associations with critically ill patients and sepsis, which are independent of AKI. NGAL, KIM-1, and IL-18 are elevated in patients with CKD. TIMP-2 and IGFBP7 have not been thoroughly tested in settings outside the ICU, and their pathophysiological roles are currently unclear. NGAL is closely associated with intrinsic AKI and displays much lower levels in pre-renal AKI, which makes them potentially suitable in the differential diagnosis of patients with established AKI (Andreucci et al., 2017; Schrezenmeier et al., 2017).

Few studies have been carried out to predict AKI using biomarkers in the context of nephrotoxicity due to the use of antibiotics. This study aimed to evaluate the role of urinary biomarkers IL-18, KIM-1, NGAL, TIMP-2, and IGFBP7 as diagnostic and prognostic predictors of AKI associated with vancomycin in hospitalized patients.

## Methods

### Study Population

This was a cohort study designed to analyze the development of AKI related to vancomycin use. It included patients 18 years of age or older admitted to the wards of Clinical Hospital from Botucatu School of Medicine and that received vancomycin. These patients were followed from the time of admission to wards until wards discharge. Nephrology fellows visited the wards daily from July 2019 to May 2020 and collected data on all patients admitted.

Exclusion criteria were chronic kidney disease (CKD) patients stages 4 and 5, kidney transplantation, wards stay <48 h, patients in the process of installing or recovering AKI of any etiology prior to the start of vancomycin, critical patients with a shock of any etiology and/or need for vasopressors, patients who were receiving others nephrotoxicity drugs as amikacin and amphotericin B. and disability in the collection of urinary samples.

**FIGURE 1:**
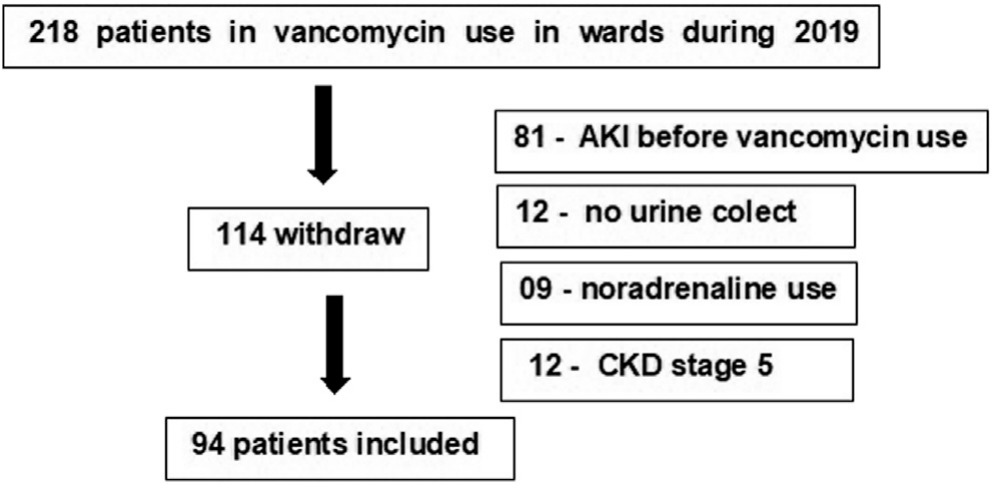
Patients undergoing vancomycin and hospitalized in wards and included in the study.

Baseline glomerular filtration rate was estimated using the clearance de creatinine estimated by the CKD EPI equation (Levey et al., 2009). Baseline creatinine was defined as the lowest serum creatinine value in the last 6 months before AKI or, for those without this measurement, the lowest value achieved during hospitalization in the absence of dialysis (Siew et al., 2010). Recovery of kidney function was defined as the return of creatinine levels to baseline value at discharge hospital moment. Figure 1 shows the inclusion and exclusion criteria.

Patient data were collected by the same observer, through consultations with the patient and their medical records, from the beginning of vancomycin until its outcome (discharge or death). Clinical (gender, age, weight, comorbidities, infectious focus, diuresis), laboratory (baseline creatinine), and vancomycin (doses, days of use, plasmatic concentrations) data were collected daily. AKI was defined and classified according to KDIGO criteria (KDIGO Acute Kidney Injury Work Group, 2012), and toxic serum vancomycin concentrations were defined as trough value higher than 20 mg/L (Rybak et al., 2009).

Dosages of uIL-18, uKIM-1, uNGAL, TIMP-2, and uIGFBP7 and plasmatic vancomycin concentrations were performed every 48–72 h from the first day of vancomycin use until the end of antibiotic therapy. All values were normalized for urinary creatinine.

This study was sent to the institution's Research Ethics Committee for analysis and started only after its approval (protocol 2,931,887).

### Biochemical Analysis

The urinary samples were centrifuged at 2,000 rpm for 5 min and stored at minus 80F0B0C and were analyzed subsequently.

Urinary IL-18, KIM-1, NGAL, TIMP-2, and IGFBP7 were measured using LUMINEX-xMAP technology at the University of Sao Paulo with reading on a MagPix device ((MerckMillipore, Austin, Texas, United States), using three kits:

• Human renal toxicity Bio-Plex Pro RBM PANEL 1 (Bio-Rad Laboratories, Berkeley, California, United States): IL-18 and KIM-1
• Human renal toxicity Bio-Plex Pro RBM PANEL 2 (Bio-Rad Laboratories, Berkeley, California, United States): NGAL
• MILLIPLEX^®^ MAP Custom Human 2-plex Magnetic Bead panel (EMD MerckMillipore, Saint Louis, Missouri, United States): TIMP-2 and IGFBP-7

Urine samples from six patients were repeated in quintuplicate to assess the reproducibility of the assays. The coefficient of variation within and between assays was 15 and 20%, respectively.

The NephroCheck^®^ kit is a rapid test in which the TIMP-2 concentration is measured multiplied by the IGFBP-7 value, and the result is divided by 1,000. As above 0.3 (ng/ml)/1,000 are positive for the test.

In addition to the dosages of BM options, we also analyzed the performance of the markers that make up the NephroCheck^®^ kit and their relationship.

### Statistical Analysis

Data analysis was performed using IBM SPSS Statistics 24.0 (United States, 2018). Continuous variables with non-normal distribution were described using median and interquartile range and those with a normal distribution as means ± standard deviation. Categorical variables were presented as *n* (%). Student’s t-test was used for data with a parametric distribution and the KruskalWallis test for non-normal data for the analysis of continuous variables. For the analysis of categorical variables, a chi-square test was used.

Diagnostic characteristics of vancomycin concentrations and urinary NGAL in predicting AKI and TIMP-2 and IGFBP-7 in predicting recovery of kidney function were assessed by the calculation of the area under the receiver operating characteristic curve (AUCROC). AUCROC analysis was performed by comparing AKI patients with all non-AKI patients and by comparing patients who recovered kidney function at hospital discharge moment with those that did not recover it. In all tests, differences were considered significant at 5%.

## Results

Ninety-four patients were included in the final analysis (Figure 1). AKI incidence was 24.5%, and it occurred 11 ± 3 days after the start of vancomycin. The mortality rate was 8.7%. Mean age was 51.1 ± 15.5 years, 67% were male, most of them had comorbidities (63.8%), and hypertension was the most frequent (in 47.9%) One hundred fourteen patients were excluded mainly due to present AKI before the introduction of vancomycin (81 patients) or due to difficulty in urine collection (Bagshaw et al., 2010) or need for noradrenalin (09 patients) or CKD stage 4 or 5. Ninety-one patients (96.8%) had vancomycin concentrations measured sequentially, and all had at least one creatinine measured during the use of the antibiotic.

Among AKI patients (23 patients), AKI KDIGO 1 occurred in 14 patients (61%). The two groups (AKI vs non-AKI) were similar in gender, length of stay, dose, and time of vancomycin use. Patients who developed AKI were older (*p* = 0.01), infection was the main cause of hospitalization (*p* = 0.02), prevalence of hypertension was higher (*p* = 0.001), plasmatic creatinine at admission was higher (*p* = 0.001), and vancomycin concentrations among 48–96 h (*p* = 0.006), 96–144 h (*p* = 0.037), 144–192 h (*p* = 0.003), and 192–240 h (*p* = 0.016) were higher, as shown in Table 1.

**TABLE 1:** Patients demographics and clinical characteristics during hopitalization at wards.

	Non-AKI *n* = 71	AKI *n* = 23	*p*
Age (years)	47.1 ± 14.5	57.13 ± 18.6	0.01
Male sex (%)	46 (64.8)	17 (74)	0.42
Creatinine at admission (mg/dl)	0.81 ± 0.24	1.09 ±0.24	0.001
Higher Creatinine (mg/dl)	0.98 ± 0.6	2.15 ± 1.46	<0.001
Creatinine at discharge (mg/dl)	0.74 ± 0.47	1.37 ± 0.86	<0.001
Comorbidities (%)			
Hypertension	20 (28.2)	15 (65.2)	0.001
Diabetes	17 (24)	10 (43.5)	0.07
CKD	4 (5.6)	1 (4.3)	0.81
Cardiovascular disease	8 (11.3)	5 (21.7)	0.20
Cancer	14 (19.7)	2 (8.7)	0.22
Cause of hospitalization (%)			
Infections	16 (22.5)	12 (50.0)	0.02
Cardiovascular	5 (7)	4 (16.6)	0.20
Surgery	38 (53.5)	5 (20.4)	0.04
Time of hospitalization (days)	34.5 ± 13.8	33.6 ± 10.94	0.9
Time of vancomycin use (days)	14.7 ± 9.6	17.3 ± 7.04	0.24
Vancomycin doses/day (g)	2.5 ± 0.9	2.24 ± 1.1	0.48
Vancomycin concentration T0-48 h (mg/L)	14.5 ± 9.3	18.3 ± 15.1	0.15
Vancomycin concentration T48-96 h (mg/L)	15.4 ± 8.9	21.7 ± 9.7	0.006
Vancomycin concentration T96-144 h (mg/L)	17.1 ± 10.1	24.2 ± 19.1	0.03
Vancomycin concentration T144-192 h (mg/L)	16.5 ± 9.1	24.3 ± 7.7	0.003
Vancomycin concentration T192-240 (mg/L)	17.5 ± 9.6	28.4 ± 18.5	0.016
Vancomycin concentration T240-288 h (mg/L)	21.3 ± 16.6	32.4 ± 27.1	0.161
Death (%)	6 (8.4)	2 (8.7)	0.97

CKD, chronic kidney disease; T, time of vancomycin use.

AKI patients had higher levels of urinary IL-18 between 144 and 192 h (*p* = 0.027), KIM-1 between 96 and 144 h (*p* = 0.005), NGAL between 96 and 144 h and 144–192 h (*p* = 0.04, *p* = 0.031, *p* = 0.012, respectively), as shown in Table 2. After normalizing the biomarkers values for urinary creatinine, there was no difference in KIM-1 levels, and AKI patients kept higher levels of urinary IL-18/creatinine between 144 and 192 h (*p* = 0,03) and urinary NGAL/creatinine between 96-144h and 144–192 h (*p* = 0.05 and *p* = 0.008, respectively) as shown in Table 3. Age (OR 6.6 CI 1.16–38.22, *p* = 0.03), vancomycin concentrations, and urinary NGAL between 96 and 144 h (OR 1.18 CI 1.04 - 1, 40 *p* = 0.04; OR 1.123, CI 1.096–1.290, *p* = 0.03, respectively) were identified as predictors of AKI at logistic regression (Table 4).

**TABLE 2:** Urinary biomarkers values according to the presence of acute kidney injury in patients hospitalized in wards and receiving vancomycin.

Urinary biomarker	Non-AKI *n* = 71	AKI *n* = 23	*p*
IL-18 (pg/ml)			
0–48 h	203.14 ± 119.27	260.45 ± 191.44	0.62
48–96 h	156.55 ± 89.8	167 ± 118.12	0.87
96–144 h	360.5 ± 20.,7	191 ± 147.8	0.7
144–192 h	98.68 ± 52.54	256.7 ± 160.11	0.027
KIM-1 (ng/ml)			
0–48 h	1.2 ± 0.13	1.3 ± 0.14	0.77
48–96 h	0.99 ± 0.22	1.1 ± 0.26	0.58
96–144 h	0.99 ± 0.23	1.8 ± 0.73	0.005
144–192 h	0.94 ± 0.1	1.05 ± 0.21	0.76
NGAL (ng/ml)			
0–48 h	262.2 ± 58.7	512.1 ± 86.1	0.09
48–96 h	229.1 ± 68.1	551.9 ± 71.9	0.03
96–144 h	374.7 ± 73.1	818.7 ± 99.8	0.03
144–192 h	252.9 ± 49.3	679.2 ± 91.5	0.012
TIMP-2 x IGFBP-7/1,000 (ng/ml)			
0–48 h	0.72 (0.18–5.01)	0.76 (0.28–5.18)	0.71
48–96 h	1.62 (0.61–5.61)	1.75 (0.07–4.46)	0.63
96–144 h	0.98 (0.48–6.30)	0.94 (0.58–5.92)	0.43
144–192 h	1.77 (0.60–4.46)	1.66 (0.082–4.09)	0.16

IL-18, interleukin 18; KIM-1, kidney injury molecule-1; NGAL, neutrophil gelatinase-associated lipocalin; TIMP-2, tissue inhibitor of metalloproteinases 2; IGFBP-7, insulin-like growth factor binding protein 7.

**TABLE 3:** Urinary biomarkers values normalized for urinary creatinine according to presence of acute kidney injury in patients hospitalized in wards and receiving vancomycin.

Urinary biomarker/creatinine (mg/dl/mg/dl)	Non-AKI *n* = 71	AKI *n* = 23	*p*
IL-18 (x 10^–7^)/cr			
1 (até 48 h)	4.56 ± 2.067	7.12 ± 1.28	0.25
2 (48–96 h)	4.95 ± 2.42	5.34 ± 1.81	0.84
3 (96–144 h)	8.92 ± 4.188	5.46 ± 2.43	0.68
4 (144–192 h)	2.44 ± 1.73	10.18 ± 6.56	0.03
KIM-1 (x 10^–7^)/cr			
1 (até 48 h)	33.68 ± 7.90	38.40 ± 4.24	0.62
2 (48–96 h)	37.30 ± 6.94	42.18 ± 6.31	0.74
3 (96–144 h)	37.08 ± 6.49	49.32 ± 6.01	0.33
4 (144–192 h)	29.24 ± 8.98	49.91 ± 8.20	0.29
NGAL (x 10^–7^)/cr			
1 (até 48 h)	69.484 ± 13.19	210.94 ± 35.86	0.05
2 (48–96 h)	181.67 ± 63.84	225.67 ± 44.60	0.008
3 (96–144 h)	191.28 ± 50.19	272.29 ± 44.86	0.55
4 (144–192 h)	36.18 ± 9.31	398.17 ± 87.13	0.05
TIMP-2 x IGFBP/cr			
1 (até 48 h)	48.11 ± 11.65	43.11 ± 29.13	0.76
2 (48–96 h)	49.21 ± 17.43	53.14 ± 31.42	0.84
3 (96–144 h)	51.17 ± 19.23	66.76 ± 41.41	0.69
4 (144–192 h)	58.16 ± 25.12	282.52 ± 66.25	0.25

IL-18, interleukin 18; KIM-1, kidney injury molecule-1; NGAL, neutrophil gelatinase-associated lipocalin; TIMP-2, tissue inhibitor of metalloproteinases 2; IGFBP-7, insulin-like growth factor binding protein 7

**TABLE 4:** Logistic Regression associated with AKI.

	Odds Ratio (95% IC)	*p*
Age	6.66 (1.16–38.2)	0.03
Vancomycin concentration (T 96–144 h)	1.19 (1.004–1.4)	0.044
Urinary NGAL (T 96–144 h)	1.123 (1.09–1.29)	0.031

AKI, acute kidney injury; NGAL, neutrophil gelatinase-associated lipocalin; T, time of vancomycin use.

AKI patients were compared in relation to the presence or absence of kidney recovery at hospital discharge, and it occurred in 12 patients (52.2%). The groups were similar in all the variables analyzed, except for the time of vancomycin use, which was longer in the group who did not recover kidney function (21.1 ± 7.6 vs. 14.16 ± 4.9 days, *p* = 0.017), as shown in Table 5. The two groups were similar in urinary biomarkers, as shown in Table 6 and Table 7. Only urinary TIMP-2 concentration multiplied by the IGFBP-7 and normalized by urinary creatinine was higher between 144 and 192 h in patients who did not recovery kidney function (*p* = 0.004 and *p* = 0.03, respectively).

**TABLE 5:** Demographics and clinical characteristics of AKI patients according to the recovery of kidney function at hospital discharge.

	Non-kidney function recovery *n* = 11	Recovery of kidney function *n* = 12	*p*
Age (years)	62 ± 19.5	52.6 ± 17.4	0.23
Male sex (%)	9 (82)	8 (67)	0.40
Creatinine at admission (mg/dl)	1.21 ± 0.67	0.98 ± 0.34	0.31
Higher creatinine (mg/dl)	2.74 ± 2.12	1.6 ± 0.6	0.08
Creatinine at discharge (mg/dl)	2 ± 0.87	0.8 ± 0.19	< 0.001
Comorbidities (%)			0.34
Hypertension	6 (54)	9 (75)	
Diabetes	4 (36)	6 (50)	
CKD	1 (9)	0	
Cause of hospitalization (%)			0.36
Infections	5 (45)	5 (42)	
Cardiovascular	3 (27)	5 (42)	
KDIGO (%)			0.31
1	5 (45)	9 (75)	
2	3 (27)	1 (8)	
3	3 (27)	2 (16)	
Time of hospitalization (days)	34.4 ± 12.19	32.9 ± 10.16	0.76
Time of vancomycin use (days)	21.1 ± 7.6	14.16 ± 4.9	0.017
Vancomycin doses/day (g)	2.1 ± 0.77	2.39 ± 1.33	0.49
Vancomycin concentration T0-48 h (mg/L)	24.7 ± 19	12.5 ± 6.6	0.47
Vancomycin concentration T48-96 h (mg/L)	22.9 ± 10.4	20.5 ± 9.2	0.58
Vancomycin concentration T96-144 h (mg/L)	25.1 ± 22.7	23.4 ± 16	0.84
Vancomycin concentration T144-192 h (mg/L)	23.3 ± 6.4	25.75 ± 9.56	0.53
Vancomycin concentration T192-240 (mg/L)	35.1 ± 18.9	15.37 ± 10.7	0.047
Death (%)	2 (18)	0	0.12

CKD, chronic kidney disease; T, time of vancomycin use.

**TABLE 6:** Urinary biomarkers value according to the recovery of kidney function at discharge hospital in AKI patients hospitalized in wards and receiving vancomycin.

Urinary biomarker	Non-kidney function recovery n = 11	Recovery of kidney function n = 12	*p*
IL-18 (pg/ml)			
0–48 h	303.14 ± 159.27	210.45 ± 191.44	0.45
48–96 h	186.55 ± 89.8	152 ± 58.12	0.74
96–144 h	183.5 ± 60.7	198 ± 37.8	0.88
144–192 h	327.68 ± 152.54	205.7 ± 60.11	0.38
KIM-1 (ng/ml)			
0–48 h	1.5 ± 0.18	1.11 ± 0.74	0.41
48–96 h	1.19 ± 0.22	1.08 ± 0.26	0.68
96–144 h	1.69 ± 0.19	1.92 ± 0.83	0.73
144–192 h	1.14 ± 0.31	1.06 ± 0.31	0.96
NGAL (ng/ml)			
0–48 h	762.2 ± 358.7	512.1 ± 86.1	0.28
48–96 h	729.1 ± 368.1	751.9 ± 271.9	0.33
96–144 h	974.7 ± 473.1	918.7 ± 399.8	0.71
144–192 h	1,252.9 ± 849.3	679.2 ± 291.5	0.17
TIMP-2 x IGFBP-7/1,000 (ng/ml)			
0–48 h	0.76 (0.18–5.01)	0.26 (0.08–1.18)	0.28
48–96 h	2.48 (0.61–5.59)	1.75 (0.07–2.46)	0.10
96–144 h	1.69 (0.88–6.30)	1.11 (0.78–3.92)	0.37
144–192 h	2.76 (1.60–4.46)	1.06 (0.08–2.09)	0.05

IL-18, interleukin 18; KIM-1, kidney injury molecule-1; NGAL, neutrophil gelatinase-associated lipocalin; TIMP-2, tissue inhibitor of metalloproteinases 2; IGFBP-7, insulin-like growth factor binding protein 7

**TABLE 7:** Urinary biomarkers values normalized for urinary creatinine according to the recovery of kidney function at discharge hospital in patients hospitalized in wards and receiving vancomycin.

Urinary biomarker/creatinine (mg/dl/mg/dl)	Non-kidney function recovery *n* = 11	Recovery of kidney function *n* = 12	*p*
IL-18 (x 10^–7^)			
1 (até 48 h)	7.91 ± 5.82	6.40 ± 1.06	0.73
2 (48–96 h)	5.14 ± 3.92	5.51 ± 3.03	0.90
3 (96–144 h)	6.97 ± 2.59	4.08 ± 3.43	0.47
4 (144–192 h)	14.41 ± 3.13	6.55 ± 2.35	0.26
KIM-1 (x 10^–7^)			
1 (até 48 h)	32,28 ± 4,80	44.01 ± 5.95	0.56
2 (48-96 h)	43,98 ± 6,70	40.67 ± 6.03	0.90
3 (96-144 h)	55,65 ± 8,12	43.52 ± 4.39	0.65
4 (144-192 h)	60,24 ± 16,89	41.07 ± 6.19	0.46
NGAL (x 10^–7^)			
1 (até 48 h)	263.68 ± 45.49	161.85 ± 31.61	0.51
2 (48–96 h)	306.70 ± 56.65	157.64 ± 26.03	0.43
3 (96–144 h)	398.52 ± 55.79	157.75 ± 20.50	0.20
4 (144–192 h)	684.27 ± 126.86	153.08 ± 20.10	0.68
TIMP-2 x IGFBP/cr			
1 (até 48 h)	62.10 ± 23.19	56.12 ± 9.49	0.28
2 (48–96 h)	74.25 ± 15.39	65.23 ± 25.19	0.10
3 (96–144 h)	78.71 ± 41.44	51.91 ± 16.84	0.37
4 (144–192 h)	22.77 ± 4.47	10.66 ± 4.44	0.03

IL-18, interleukin 18; KIM-1, kidney injury molecule-1; NGAL, neutrophil gelatinase-associated lipocalin; TIMP-2, tissue inhibitor of metalloproteinases 2; IGFBP-7, insulin-like growth factor binding protein 7

Logistic regression identified non-recovery of kidney function after AKI, the time of vancomycin use (OR 4.6, CI 1.11–22.02, *p* = 0.03), TIMP-2 multiplied by IGFBP -7 between 144 and 192 h normalized by urinary creatinine as predictors (OR 1.26, CI 1.092–1.543, *p* = 0.03), as shown in Table 8.

**TABLE 8:** Logistic Regression associated with recovery of kidney function.

	Odds Ratio (95% IC)	*p*
Time of vancomycin use (days)	4.61 (1.11–22.02)	0.03
uTIMP-2xIGFBP-7/cr (144–192 h)	1.26 (1.09–1.54)	0.03

**FIGURE 2:**
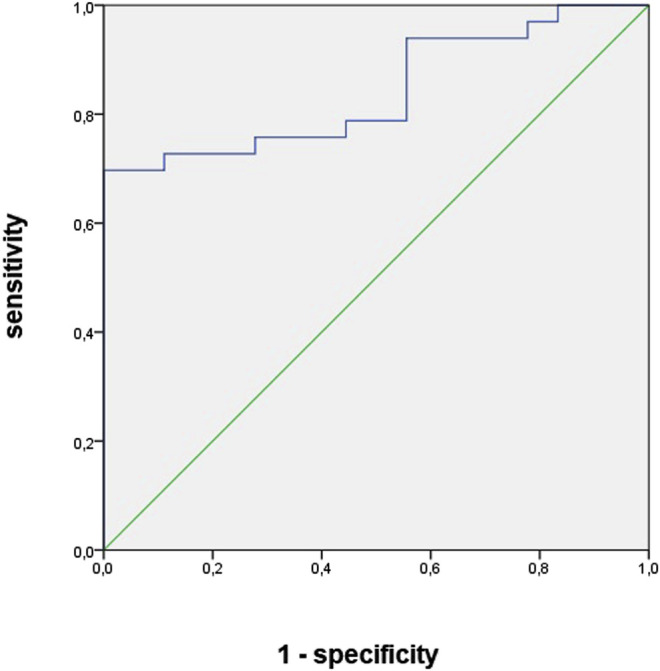
Area under curve of vancomycin concentration as predictors of acute kidney injury diagnosis in patients undergoing vancomycin and hospitalized in wards.

**FIGURE 3:**
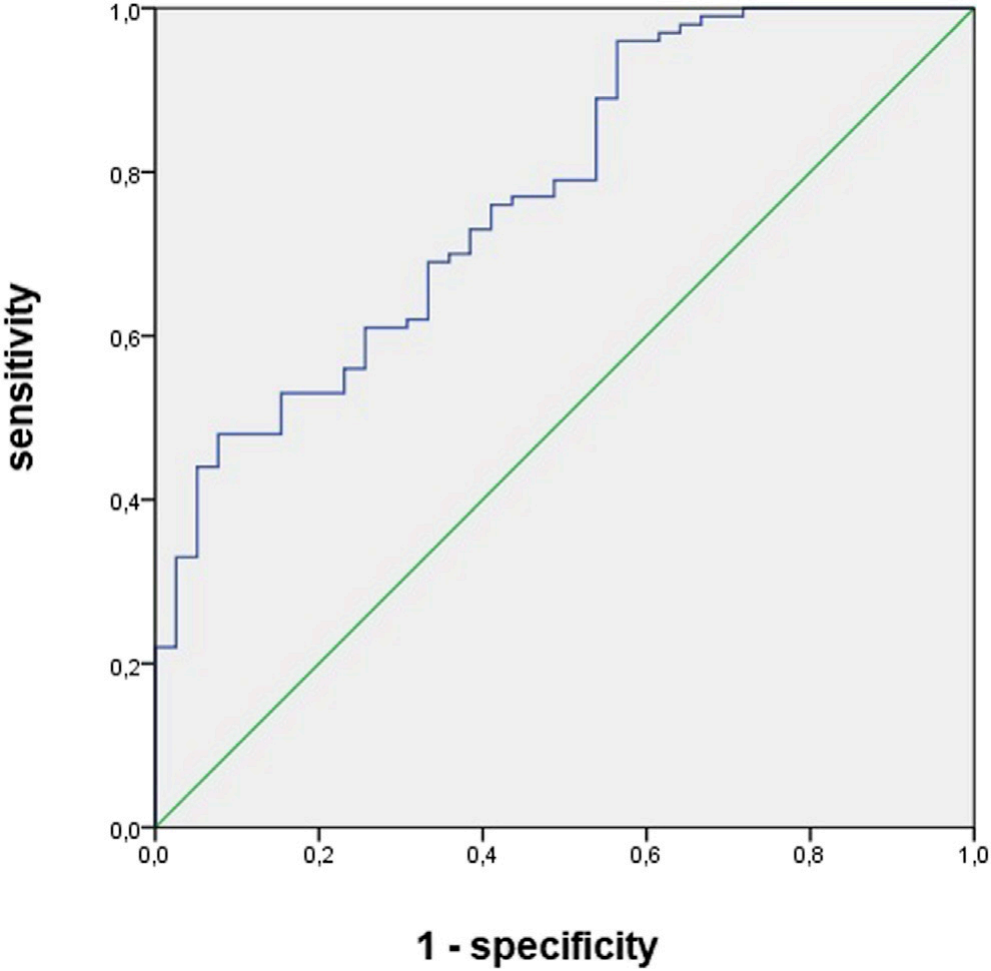
Area under curve of urinary NGAL between 144 and 192 h as predictors of acute kidney injury diagnosis in patients undergoing vancomycin and hospitalized in wards.

The areas under the curve for vancomycin concentration and urinary NGAL levels between 96 and 144 h were 0.76 and 0.82, respectively. Both plasmatic vancomycin concentrations and urNGAL were good predictors of AKI within the next 5 days. The optimal cutoff value of them had sensitivity and specificity of 0.79 and 0.66, 0.83 and 0.68, respectively (Table 9, Figure 2 and Figure 3).

**TABLE 9:** Sensitivity, specificity, area under curve, cutoff values of urinary biomarkers, and vancomycin concentration as predictors of diagnosis and recovery of kidney function in patients undergoing vancomycin and hospitalized in wards.

	AUC	*p*-value	*Cutoff*	Sens	Spe	IC
*AKI diagnosis*						
Vancomycin concentration (T 96–144 h)	0.76	0.03	23.8	0.69	0.66	0.71–0.98
Urinary NGAL (T 96–144 h)	0.82	0.02	618.8	0.73	0.68	0.61–0.96
*Recovery of kidney function*						
Urinary TIMP-2xIGFBP-7/1,000 (144–192 h)	0.71	0.009	2.15	0.88	0.64	0.62–0.98

AUC, area under curve; sens, sensitivity; spe, specificity.

**FIGURE 4:**
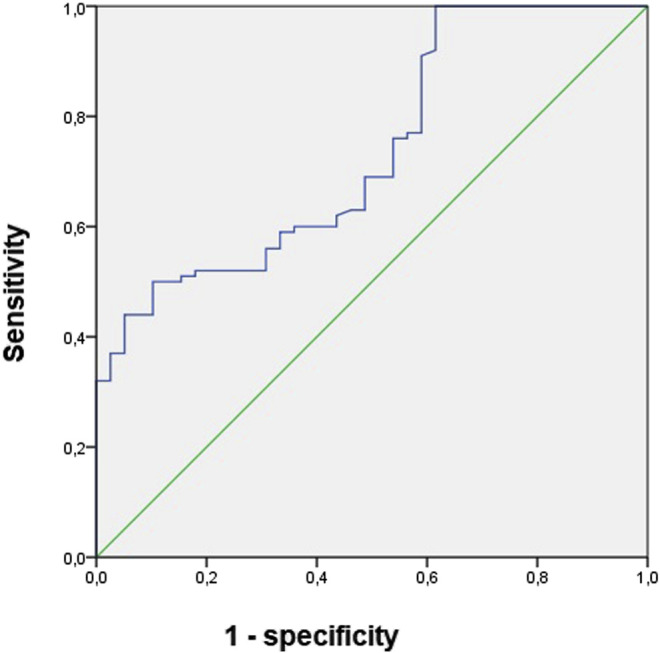
Area under curve of TIMP-2 multiplied by IGFBP-7 and normalized by urinary creatinine between 144 and 192 h as predictors of recovery of kidney function in patients undergoing vancomycin and hospitalized in wards.

Concerning urinary biomarkers of cell cycle arrest as predictors of recovery of kidney function at discharge hospital, the area under the curve for TIMP-2 multiplied by IGFBP-7 and normalized by urinary creatinine between 144 and 192 h was 0.71, (Table 7, Figure 4). The optimal cutoff value was 0.3, and sensibility and specificity were 0.88 and 0.64.

## Discussion

AKI is a common and important condition to be recognized, depending on the serum creatinine for its diagnosis. Unfortunately, creatinine is a late and unreliable biomarker. According to Ostermann et al., there is a persistent unmet need for an earlier identification of patients with AKI. Furthermore, diagnostic tools that identify the location, mechanism, etiology, severity, and prognosis of AKI are necessary and have stimulated studies on new biomarkers, among them the IL-18, NGAL, KIM-1, TIMP-2, and IGFBP-7, identifying promising predictors of AKI in different scenarios such as cardiac surgery and sepsis (Wagener et al., 2006; Hirsch et al., 2007; Bagshaw et al., 2010; Liu et al., 2013; Singa et al., 2015; Luo et al., 2016; Mcduffie et al., 2016; Rocha et al., 2018; Ostermann et al., 2020); however, few studies evaluated the role of urinary biomarkers in AKI associated with vancomycin nephrotoxicity.

Despite the widespread use of vancomycin in practice, concerns related to its safety still permeate the academic environment. Emphasis has been given to its nephrotoxicity, with the risk of AKI with short- and long-term complications. Ninety-four patients were included while 114 were excluded, mainly because they already had AKI before using vancomycin (81) or because of the difficulty in urinary collection (Funk et al., 2016), which reflects the difficulty in studying the role of vancomycin nephrotoxicity, leading to doubt whether the high serum vancomycin concentrations are the cause or the consequence of AKI (Bosso et al., 2011; Elyasi et al., 2012; Hanrahan et al., 2014; Zamoner et al., 2020).

Among the population studied, the incidence of AKI was 24.5%, with a predominance of KDIGO 1 (61%) and mortality of 8.7%, which indicates that we really studied without AKI associated with sepsis, in which mortality is much higher, around 35% (Srisawat and Kellum, 2011). Data from the previous studies suggest variable nephrotoxicity prevalence, from 5 to 40% in the presence of other nephrotoxic drugs (Elyasi et al., 2012; Hanrahan et al., 2014; Pazhayattil and Shirali, 2014; Funk et al., 2016; Zamoner et al., 2020).

At logistic regression, only older age, higher creatinine at admission, and concentrations of vancomycin and urinary NGAL between 96 and 144 h were factors associated with AKI.

According to several studies, the age of patients remains as one of the most important risk factors for AKI, due to kidney senility, high frequency of comorbidities, and several medical procedures (Funk et al., 2016; Petronijevic et al., 2017).

Serum concentration >23.8 mg/L and u NGAL >618 ng/ml showed to be good predictors of AKI, with an area under the curve of 0.76 and 0.82, the sensitivity of 79% and 83%, the specificity of 66% and 68%, respectively, and both of them preceded the diagnosis of AKI by 5 days (AKI happened on average on the eleventh day of vancomycin use).

Bosso et al. (Hirsch et al., 2007) performed a study that evaluated 288 patients using vancomycin and showed an association between nephrotoxicity incidence and vancomycin concentrations, with AKI occurring in almost 30% of patients with plasmatic concentrations of vancomycin >15 mg/L and 9% of patients with vancomycin concentrations <15 mg/L. Recently, Zamoner et al. (2020) found that plasmatic concentration of vancomycin was an excellent predictor of AKI in patients admitted to wards, preceding the diagnosis of AKI by at least 72 h.

The two urinary biomarkers studied most often for early diagnosis of AKI have been urine IL-18 and NGAL, mainly in sepsis and patients undergoing cardiac surgery. NGAL performed very well in two specific populations: children undergoing cardiac surgery and patients receiving kidney transplantation; however, it did not perform as well in critically ill children or adults and in adults undergoing cardiac surgery (Levey et al., 2009; Schiffl and Lang, 2012; Liu et al., 2013; Ronco, 2014).

The AUCs for IL-18 in four studies varied markedly (0.54–0.9). In general, IL-18 showed low sensitivity but high specificity for the early diagnosis of AKI. Similar to NGAL, the performance of IL-18 is dependent on the time of collection in relation to the exposure (such as surgeries) and the AKI (Leslie and Meldrum, 2008). In summary, increases in urine IL-18 concentration are rarely false-positive elevations; however, several AKI patients do not have increased in urine IL-18.

Concerning KIM-1, it appears that it does not have good accuracy in early prediction of AKI, but it is good in identifying established AKI (Huang and Craig Don-Wauchope, 2011).

Regarding the non-recovery of renal function until the moment of hospital discharge, logistic regression identified the time of use of vancomycin and the highest TIMP-2 multiplied by IGFBP-7 between eighth and tenth days of antibiotic use as factors associated with non-recovery of renal function.

The area under the curve for TIMP-2 multiplied by IGFBP-7 was 0.71, the optimal cutoff value was 2.35, and sensibility and specificity were 0.88 and 0.64.

To date, we are unaware of previous studies that have identified the length of use of vancomycin as a predictor of AKI prognosis.

Some biomarkers mentioned above were evaluated not only for AKI diagnosis but also for its prognosis, such as the recovery of kidney function.

TIMP-2 and IGFBP-7 have a direct role in cell cycle arrest mechanisms, and both proteins are expressed in tubular cells in response to DNA damage and possibly other forms of damage. The exact biological role of these proteins in AKI, in addition to their usefulness as biomarkers, is not fully understood (Ortega and Heung, 2018). Its levels are believed to decrease in the face of cell repair.

Ostermann et al. (2018), in an ancillary analysis of the multicenter SAPPHIRE study, examined the kinetics of the urinary TIMP-2 * IGFBP-7 in association with exposure to common renal insults (major surgery, IV radiocontrast, vancomycin, nonsteroidal anti-inflammatory, and piperacillin/tazobactam). The authors compared the urinary TIMP-2 * IGFBP-7 kinetics from the day prior to exposure up to 5 days after exposure in patients developing AKI stage 2–3, stage 1, or no AKI. Among the 723 patients, 679 (94%) had at least one, 70% had more than one, and 35% had three or more exposures to a known renal insult. There was a significant association between a cumulative number of exposures up to study day 3 and risk of AKI (*p* = 0.02) but no association between the specific type of exposure and AKI (*p* = 0.22). With the exception of radiocontrast, patients who developed AKI stage 2–3 after one of the five exposures had a clear rise and fall of urinary TIMP-2 * IGFBP-7.

The Sapphire study evaluated AKI prognosis, which includes death, need for dialysis, or persistence of renal dysfunction (creatinine above 2 times baseline) after 30 days (MAKE 30). The risk for MAKE 30 was lower when TIMP-2 • IGFBP7 <0.3 (18%), increasing when TIMP-2 • IGFBP7 between 0.3 and 2 (23%), and high when TIMP -2 • IGFBP7] 2 (40%); *p* < 0.001 (Kashani et al., 2013).

In this study, the biomarkers KIM-1 and IL-18 did not play a relevant role as diagnostic or prognostic predictors of AKI associated with vancomycin.

Our study presents some limitations. First, the sample obtained was small, reflecting the difficulty of studying nephrotoxicity. Second, the data were obtained from a single center. Third, there was absence of long-term follow-up, and finally, the role of plasmatic vancomycin concentration and urinary biomarkers was not studied as a prognostic predictor of AKI severity, of need for acute renal support, and of death due to the small number of events.

Despite these limitations, this is the first study to present cutoff of urinary biomarkers with the objective of evaluating their role as diagnostic and prognostic predictors of AKI associated with vancomycin.

## Conclusion

Age, vancomycin concentrations, and urinary NGAL levels between 96 and 144 h are predictors of AKI, anticipating its diagnosis in at least 5 days, while the time of vancomycin use and urinary levels of TIMP-2. IGFBP-7 normalized by urinary creatinine between sixth and eighth day of vancomycin use were associated with TIMP-2 non-recovery of kidney function among patients with AKI at the time of hospital discharge, indicating the possible evolution to chronic kidney disease. More and larger studies are needed to clarify the role of biomarkers in AKI associated with vancomycin.

## Data Availability

The raw data supporting the conclusions of this article will be made available by the authors, without undue reservation.
